# Discovery of Novel Functional Centers With Rationally Designed Amino Acid Motifs

**DOI:** 10.1016/j.csbj.2018.02.007

**Published:** 2018-02-27

**Authors:** Aloysius Wong, Xuechen Tian, Chris Gehring, Claudius Marondedze

**Affiliations:** a Department of Biology, Wenzhou-Kean University, 88 Daxue Road, Ouhai, Wenzhou, Zhejiang Province 325060, China; b Department of Chemistry, Biology & Biotechnology, University of Perugia, Borgo XX giugno, 74, 06121 Perugia, Italy; c Laboratoire de Physiologie Cellulaire et Végétale, Université Grenoble Alpes, CEA/DRF/BIG, INRA UMR1417, CNRS UMR5168, 38054 Grenoble Cedex 9, France

**Keywords:** Search motif, Functional centers, Hidden domains, Structural modeling, Molecular docking

## Abstract

Plants are constantly exposed to environmental stresses and in part due to their sessile nature, they have evolved signal perception and adaptive strategies that are distinct from those of other eukaryotes. This is reflected at the cellular level where receptors and signalling molecules cannot be identified using standard homology-based searches querying with proteins from prokaryotes and other eukaryotes. One of the reasons for this is the complex domain architecture of receptor molecules. In order to discover hidden plant signalling molecules, we have developed a motif-based approach designed specifically for the identification of functional centers in plant molecules. This has made possible the discovery of novel components involved in signalling and stimulus-response pathways; the molecules include cyclic nucleotide cyclases, a nitric oxide sensor and a novel target for the hormone abscisic acid. Here, we describe the major steps of the method and illustrate it with recent and experimentally confirmed molecules as examples. We foresee that carefully curated search motifs supported by structural and bioinformatic assessments will uncover many more structural and functional aspects, particularly of signalling molecules.

## 1 Introduction

Plant biotechnological innovations and genetic engineering require an understanding of the signalling pathways in plant cells including their constituent molecular components which are often complex in nature [[1], [2], [3]]. As sessile organisms, plants are constantly exposed to fluctuations in environmental conditions and stresses including high salinity, temperature, light and pathogens that challenge their growth, development and reproductive capabilities [[4], [5], [6]]. In response to these stresses they have evolved adaptive strategies including rapid and effective molecular signal perception and processing that in many instances are distinct from those in animals [[7], [8], [9], [10], [11], [12]]. Yet, they also make use of signalling molecules that exist in many prokaryotic and lower eukaryotic cells leveraging on the ubiquitous second messengers such as calcium ions [13], cyclic nucleotide monophosphates i.e., cyclic guanosine 3′,5′‑monophosphate (cGMP) and cyclic adenosine 3′,5′‑monophosphate (cAMP) [[14], [15], [16], [17]], and the gaseous nitric oxide (NO) [[18], [19], [20], [21], [22]] in addition to generating their own set of plant specific hormones [3] to perceive environmental cues, transduce external signals into the cell and orchestrate appropriate responses. Since the release of the *Arabidopsis thaliana* genome – a model organism for plant research – in 2000 [23], a wealth of molecular data and information have become available many of which are based on orthologues in animals, fungi and bacteria [[24], [25], [26]]. However, regular homology-based approaches did not yield a comprehensive coverage of the signalling pathways as many molecules known to perform key signalling roles in other eukaryotic cells, are seemingly elusive in the plant cell [15,27,28]. This is because rather than a stand-alone molecule, many plant proteins have evolved complex domain organizations [29,30] consisting for instance of an extracellular ligand recognition receptor region, a single or multi-pass transmembrane region and a cytosolic region that may accommodate one or indeed several functional domains with protein-protein interaction or catalytic roles [31,32]. Thus, their corresponding functional region is masked by a much larger multi-domain protein [33] and made more complicated still if they have diverged beyond the detection limits of BLAST and antibodies raised in animals or bacteria [34]. The differences between the plant and animal cellular signalling environments and mechanisms are at least in part due to the crowded cellular environment and rigid temporal and spatial distribution of signalling components in plant cells [33,[35], [36], [37], [38], [39]] that unlike in animal cells, are occupied by large vacuoles.

**Fig. 1:**
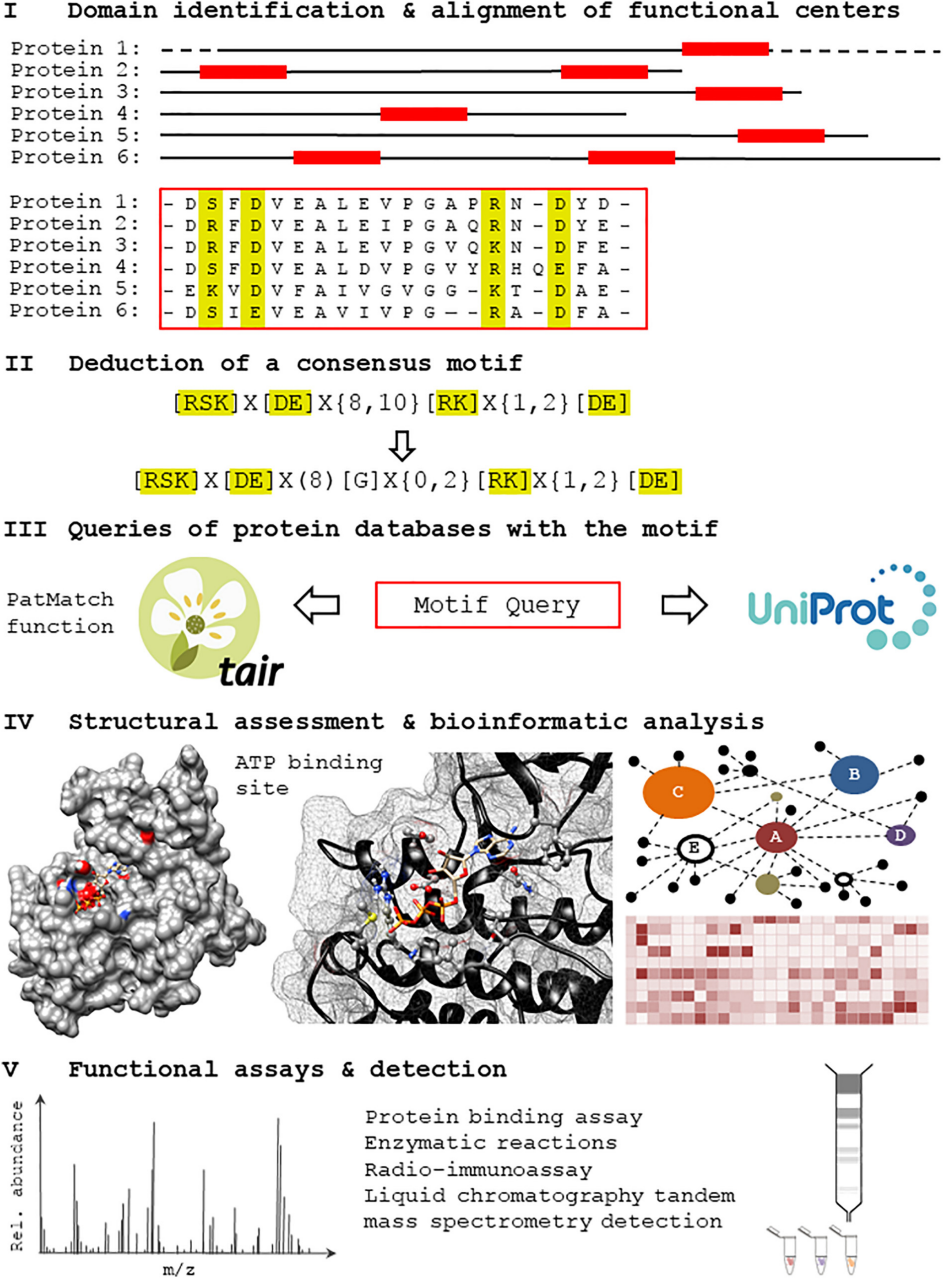
An Illustration of a motif-based approach discovery of functional centers. In step I, annotated functional centers (red blocks – in this example we look for the consensus sequence of an AC catalytic center) in known domains from organisms represented across species are aligned to allow for subsequent consensus motif building in step II. The consensus motif is then searched against protein databases such as UniProt (http://www.uniprot.org) or organism-specific databases such as TAIR (https://www.arabidopsis.org) in step III. If the retrieved candidate numbers are too high, additional ancillary residues can be added to increase specificity or if the candidate numbers are too low the motif can be relaxed (back to step II). Once a workable list of candidates is obtained, the proteins are subjected to structural evaluations and bioinformatic analysis (step IV) making use of information from publicly available databases to order the list based on both structural and biological interest. The top candidates can then be selected e.g. for the synthesis of recombinant proteins or fragments that in turn can be assayed in *in vitro* by high resolution detection methods (step V).

Given the structural differences of the peptide signalling components in plants, a motif-based approach has been implemented specifically for the identification of functional centers in complex multi-domain plant molecules and has led to the discovery of molecules such as nucleotide cyclases (guanylate cyclases (GCs) and adenylate cyclases (ACs)), NO sensing molecules, and molecules directly modulated by the hormone abscisic acid (ABA). Here, we define ‘functional center’ as a region of a peptide sequence within a protein, typically smaller than a regular domain that exist as distinct functional site or embedded within a larger domain. The amino acids in the functional center perform a specific molecular function including but not limited to catalysis, ligand-binding or protein-protein interaction. Our computational approach is based on a broad survey of molecules of a particular function (e.g. GCs or ACs) across kingdoms with a view to identify such functional centers in plants and assess if they are conceivably operating in plants too. But unlike homology-based approaches, only amino acid residues that have direct roles critical in performing the molecular function are included in the construction of an amino acid search motif [32,39]. This approach is based on the assumption that key amino acid residues directly involved in performing a molecular function at e.g., a catalytic center or a ligand binding site, are highly conserved across species, while intermediary and flanking residues may be less conserved since many different amino acid combinations can assume similar structural folds. The approach consists of a number of distinct steps. Firstly, an alignment of functional centers in known domains of molecules across species is performed where highly conserved amino acid residues with annotated molecular functions are included in an initial search motif. Secondly, this motif can be subjected to rational modifications (made more or less stringent), where residues of similar size and charge which could conceivably perform the same function as the amino acid residue in that particular position of the motif, can be included. Thirdly, the curated motif is then used to search for candidates in the Arabidopsis proteome using pattern matching tool (PatMatch) feature [40] in the Arabidopsis Information Resource (TAIR) website (https://www.arabidopsis.org). If the list of hits is overly exhaustive, the motif can be further curated to include amino acid residues that are known to regulate or selectively enhance the primary function. Fourthly, once a reasonable list of candidates (>50) is obtained, the next phase of screening includes structural assessments in the form of homology modeling and ligand docking simulations of selected candidates using software such as Modeller [41] and AutoDock Vina [42]. The list can now be ordered based on the results of this structural evaluation. Additionally, top candidates are further subjected to a systematic bioinformatics analysis extracting data from publicly available databases to infer biological functions [43]. For instance, knowledge obtained from the investigation of genes whose expressions are co-related to the selected candidate gene may reveal information about its interacting partners, cellular localization, tissue specificity and expression patterns at different developmental stages, as well as its expression patterns under selected biotic and abiotic cues. Finally, these computational assessments will serve to select candidates for *in vitro* testing with methods of high resolution and sensitivity [44,45]. An illustration of this workflow is shown as Fig. 1. The experimental data will then be used to revisit and further refine the search motifs and, if the functional tests have been positive, to assess how such centers look in orthologues and paralogues.

In this review, we highlight the implementation of this motif-based approach citing recent and experimentally confirmed molecules as examples. We put the emphasis on the discovery of plant nucleotide cyclases, enzymes that generate cyclic nucleotide monophosphates, NO-sensing molecules and the recent identification and characterization of novel ABA binding sites. In addition, we discuss how systematically curated search motifs can be generated for the discovery of hitherto elusive functional centers.

## 2 Nucleotide Cyclase Functional Centers in Plants

This motif-based approach was first applied in a search for cGMP-generating enzymes, (GCs) in plants where previous searches with GCs from cyanobacteria, fungi and animals failed to identify candidates in *Arabidopsis thaliana* [34,46] although a GC-like gene that is associated with Gibberella ear rot resistance from *Zea mays* [47] and a canonical GC from *Hippeastrum hybridum* that is functional and is responsive to wounding and pathogen infection, have since been reported [48]. Cyclic nucleotide monophosphates in particular cGMP, have been implicated in a broad range of plant responses e.g. to light [49], hormones [[50], [51], [52]], salt and drought [53,54], ozone [55,56], pathogens [14,[57], [58], [59]] and signalling peptides [60,61]. Cyclic nucleotide monophosphates are also gating ion channels in particular, the cyclic nucleotide-gated channels (CNGCs) [62], resulting in a direct regulation of cellular ion homeostasis. Since cGMP is detected in *Arabidopsis thaliana* [44,59,63] and its biological functions have been well-established [16,17,[64], [65], [66], [67]], the generating enzymes (GCs) must therefore exist but are most likely not orthologues of GCs in animals, fungi or bacteria but may contain conserved catalytic centers that may be part of multi-domain proteins [15,31,68]. For this reason, we propose that functional GC centers identified from this motif-based approach constitute a new class of GCs [68] that are often found in complex multi-domain proteins with primary functions such as ligand-recognition sites, gas sensors or kinases, and are thus different from canonical GCs.

A 14-amino acid long GC search motif ([RKS][YFW][CTGH][VIL][FV]G[DNA]X[VIL]X{4}[KR]) [34] was first deduced from the alignment of designated catalytic regions from prokaryotes and eukaryotes. Position 1 of this GC motif contains amino acids that form hydrogen bonds with guanine of the substrate GTP [69,70] and amino acids in position 3 confer specificity to GTP thus enabling GCs to discriminate GTP from ATP [71]. Amino acids in position 14 of the motif bind to the phosphate acyl group and stabilize the transition of GTP to cGMP [69]. This GC motif was used in a PatMatch search against the Arabidopsis proteome and identified the first GC (AtGC1) in higher plants which generated >20 fmol cGMP per μg protein *in vitro* as detected by radioimmunoassay [34]. In 2009, a protein in morning glory (*Pharbitis nil*), PnGC-1, that has high homology to known plant GCs was shown to be a functional GC *in vitro* [72]. Furthermore, the transcripts of *PnGC-1* are regulated by light thus suggesting a potential role of the GC in light signal transduction [72]. Although PnGC-1 does not harbour the full 14-amino acid GC motif, it however contains the conserved functional amino acids in the GC motif ([KS]X[CGS]X{10}[KR]) as described in [31], at position S5 – K18 of the protein. The GC motif and its derivatives have further identified an additional four molecules containing functional GC centers in *Arabidopsis thaliana* where they are embedded within larger kinase (or kinase-like) domains of a wall-associated kinase-like protein (AtWAKL10) [73], the brassinosteroid receptor (AtBRI1) [74], the PepR1 receptor (AtPepR1) [75] and the phytosulfokine receptor (AtPSKR1) [50]. Later, an additional GC center was identified within an Arabidopsis natriuretic peptide (PNP) receptor (AtPNP-R1) [61]. An orthologous PepR1 protein from *Hippeastrum hybridum* was also shown to generate cGMP *in vitro* and to be responsive to pathogen infection and wounding [76]. A more “relaxed” GC motif that includes a serine residue at position 3, converting a thioester configuration into an ester configuration, has led to the identification of an NO-regulated GC (AtNOGC1) [77]. Experimental confirmation of new candidates will contribute to the development and refinement of the GC motif and this iterative process will in turn, improve our understanding of the nature of this class of GCs. Subsequent step-wise rational modifications of the GC search motif have enabled the identification of a large number of candidate GCs in the Arabidopsis proteome [17,31] that may exist in combinations with other domains such as the Heme NO/OXygen (H-NOX), GAF-like, protein kinase-like and ATPase domains [68]. This finding may not be surprising considering that such diverse multi-domain configurations also exist in a unicellular green alga, *Chlamydomonas reinhardtii* [34].

More recently, ACs, the enzymes that generate a closely related signalling molecule, cAMP, have also been identified using a similar approach [15]. Much like cGMP, cAMP has also been detected in many plants including *Arabidopsis thaliana* [44]. In general, in eukaryotic cells cGMP and cAMP were reported to mediate a number of different biological responses including effects of hormones [78], the regulation of signalling pathways critical for adaptation and survival [[79], [80], [81]], stress and defense responses [82,83] and more specifically, the activation of protein kinases [84] and promotion of cell division [85]. Cyclic AMP also causes stomatal opening [86] and modulates ion transport through the CNGCs [87,88]. Apart from a *Zea mays* AC that participates in polarised pollen tube growth [89], there were no previous reports of plant ACs. By substituting the amino acid at position 3 of the GC motif to change substrate specificity from GTP to ATP (i.e., [CGTH] → [DE]) [71]), an AC motif was created and this motif has identified candidates harboring this AC center. One candidate, a K^+^ transporter, AtKUP7 can generated >40 fmol/μg protein of cAMP *in vitro* as detected by mass spectrometry [90]. Importantly, recombinant AtKUP7 was also able to complement the AC-deficient mutant *cyaA* in *Escherichia coli* [90].

This method of identification was possible because the catalytic centres of ACs and GCs differ only in the amino acid that confers substrate specificity and native GCs have been previously shown to be able to assume the catalytic function of ACs and *vice versa* when this amino acid residue implicated in substrate recognition was mutated [71,91]. This GC-derived AC motif has further identified a functional AC center in a pentatricopeptide repeat-containing protein (AtPPR) [92] and predicted several candidate ACs in *Arabidopsis thaliana* [15,17] some of which are currently under experimental evaluation. Recently, a functional AC domain that contains the AC catalytic center motif has been reported in liverwort *Marchantia polymorpha* and interestingly, this protein also harbors a phosphodiesterase (PDE) domain that is capable of degrading cyclic nucleotide monophosphates [93] thus representing an example of a complex multi-domain plant protein capable of regulating cellular cyclic nucleotide monophosphates levels. However, this protein has orthologues only in basal land plants and charophytes that use motile sperms as the male gamete [93] and thus a *bona fide* PDE-AC in higher plants remains elusive.

The use of a search motif for the identification of functional GC and AC centers is strengthened by concomitant structural evaluations [31,39] especially when good template structures are available for the generation of reliable 3D models. This structural approach that includes docking simulations of ligands to the catalytic centers, has been applied in the discovery of the AC center in AtKUP7 [90] and used to guide mutation analyses in AtBRI1 [52]. Notably, computational simulations of previously characterized GC centers have established the GTP binding pose and orientations at the catalytic centers at the same time revealing detailed atomic level information about the interactions between substrate and key amino acids within the catalytic centers [39]. Importantly, probing of 3D models also revealed consistent secondary structures at the catalytic centers across all plant GCs characterized to-date [67]. This computational approach has led to the discovery of functional centers that are dissimilar in architecture to the animal and bacteria cyclases. The identified nucleotide cyclases have been proposed to constitute a new class of plant nucleotide cyclases that typically reside within larger domains of proteins [16,68]. These embedded functional centers have yield consistently lower amounts of cyclic nucleotide monophosphates than their animal counterparts ranging from 20 fmol cGMP/μg protein in AtGC1 [34] to 699 fmol cGMP/μg protein in AtPNP-R1 [61] while AtKUP7 generated 42.5 fmol cAMP/μg protein [90]. Comparisons with the amounts obtained from other organisms are only natural given their biological significance [94,95] and as such, the use of high resolution detection methods e.g. tandem liquid chromatography mass spectrometry (LC-MS/MS) are encouraged in addition to using enzyme and radio immuno- assays to ascertain *bona fide* catalytic activities. An optimized LC-MS/MS method [45] that is capable of detecting as little as 5 fmol/μL cyclic nucleotide monophosphates (detectable above background) has been developed. This highly sensitive method has detected and quantified cyclic nucleotide monophosphates generated by AtBRI1 [52] and AtKUP7 [90] because quantification relies on the use of daughter peaks *m/z* 136 [M + H]^+^ and *m/z* 152.06 [M + H]^+^ that resulted from the fragmentations of their parent cGMP and cAMP precursor ions, respectively, in a second ionization step [67]. Since the fragmented product ions are unique to their parent compounds, this approach reliably discriminates cyclic nucleotide monophosphates generated from recombinant proteins and from those in e.g. un-induced bacterial extracts, therefore unequivocally confirming the catalytic capabilities of the respective AC or GC centers [67]. The use of less sensitive detection approaches such as UV-based spectrometry and sub-optimal enzymatic conditions, can be the cause of false negative results.

Naturally, concerns about whether typically low amounts of cyclic nucleotide monophosphates from plant nucleotide cyclase centers can conceivably carry any biological significance were raised [95] and discussed [94]. In recent years, experimental data demonstrated a clear link between the GC centers and the immediate signalling pathways as well as the physiological implications [17]. At the molecular level, the GC activity of AtBRI1 requires a functional kinase domain while the generated cGMP product, in turn inhibits BRI1 kinase activity and potentiates phosphorylation of downstream substrates including the brassinosteroid signalling kinase 1 (BSK1), consequently assigning a modulatory role for cGMP [52]. In another similar receptor complex AtPSKR1, Ca^2+^ was determined as a bimodal molecular switch that selectively enhances the GC activity while inhibiting the kinase activity [38]. In addition to intrinsic micro-regulations between the primary and its moonlighting nucleotide cyclase centers at the cytosolic regions of AtBRI1 and AtPSKR1 [33,37], binding of the extracellular receptor domains to their natural ligands are capable of elevating intracellular cGMP levels thus implying physiological functions [50]. Similarly, binding of plant natriuretic peptide (PNP) to its receptor AtPNP-R1 is essential for PNP-dependent regulation of ion and water homeostasis as observed from studies in protoplasts of wildtype and knockdown mutant plants [61]. Further evidence of biological significance can also be seen from work on PepR1 of *Hippeastrum hybridum* where transcript levels augmented in response to *Peyronellaea curtisii* fungal infection but not to mechanical wounding implicating cGMP-dependent signalling in rapid plant responses to pathogen infection [76]. Another molecule with a functional GC center in morning glory (*Pharbitis nil*), PnGC1, has transcript level modulated by different light regimes [72].

## 3 Nitric Oxide Sensors in Plants

A motif-based approach was also applied to identify NO-sensing molecules in plants. The gaseous NO is a key signalling molecule involved in many plant developmental processes [21,96,97] and fertilization particularly in tip-growing cells [20,22,[98], [99], [100]]. In the absence of canonical cellular receptors, recognition of this rapidly diffusing signal by molecular sensors is therefore crucial for NO-dependent plant responses and these molecules are surprisingly elusive. Here, we define “sensors” as molecules with the ability to preferentially bind NO in a reversible manner and typically triggering a change in molecular functions leading to a cellular and/or biological response. The alignment of heme centers of functional gas-responsive heme-NO/oxygen [101], heme-NO binding [102] and sensor of NO [103] family of proteins from prokaryotes and eukaryotes, allowed for the construction of a NO-sensing H-NOX search motif (HX{12}PX{14,16}YXSXR) [22,77]. The first amino acid of this pattern, the histidine, coordinates to the heme group's central iron atom as an axial ligand where it was previously shown that the iron center is responsible for reversible NO-binding [104] while the conserved proximal proline induces a steric strain at the bound heme cofactor facilitating the breaking of the dative histidine-iron bond [105]. The downstream ‘YXSXR’ signature forms hydrogen bonds with the carboxylates of the heme *b* and thus enhances the affinity of the prosthetic group [106]. This motif retrieved four candidates in *Arabidopsis thaliana,* one of which, AtNOGC1 has been shown to bind NO with higher affinity than O_2_ and binding to NO activated the GC catalytic center that was also identified from a GC search motif [77]. A biological implication was later ascertained when the NO-dependent and cGMP-mediated stomatal closure was impaired in *atnogc1* mutant plants [107]. Interestingly, another H-NOX molecule AtDGK4 [22], is expressed only in the pollen and previous evidence of NO-dependent processes in pollen such as pollen tube re-orientation, pollen-stigma interactions, guidance of the growing tube to the ovule [[108], [109], [110]] as well as actin organization, vesicle trafficking and cell wall deposition [97], has invited speculation that these biological processes are, at least in part, dependent on sensing of NO by AtDGK4.

## 4 ABA Modulatory Sites

It was recently reported that the conductance of *Arabidopsis thaliana* guard cell outward-rectifying potassium, AtGORK, is directly modulated by ABA [111], and subsequent amino acid sequence analysis and structural probing of AtGORK has revealed a cytosolic region that harbors amino acid residues reminiscent of the latch-like region of ABA-binding sites of the PYR/PYL/RCARs which are ABA receptors known to function via a conserved gate-latch-lock mechanism [112]. Guided by structural evaluations, this region of AtGORK was determined by immunoassay to have high affinity for ABA that was reduced when the conserved amino acids (K559 and Y562) were mutated. Functional studies of AtGORK in a heterologous system demonstrated activation of this channel by ABA while the same amino acid mutations reduced the ABA-dependent activation [111]. Since this molecule is primarily a potassium channel and only retains some conserved residues of known canonical ABA receptors, the characterized region is therefore likely a modulatory site. The authors explained that the binding of ABA to this site enables rapid stomatal regulation through the direct enhancement of K^+^-efflux via GORK – a mechanism that allows plants to efficiently adapt to environmental stresses [111].

Given these findings and the literature that reported two GPCR-type G proteins as ABA receptors [113], it appears possible that multi-domain plant proteins with ABA interacting and modulatory sites await discovery. This notion is also supported by earlier biochemical evidence that predicts the presence of both cell-surface and intracellular receptors for ABA [114]. We have therefore deduced an “ABA interacting” search motif ([DE]X{7,8}RX{3,4}[DE]X{5}YX{6}H) by including the conserved amino acid residues in the latch-like region of canonical ABA receptors and ABA interacting AtGORK (see Fig. 1A in [111]) to identify ABA modulatory sites within complex multi-domain plant proteins. This motif further identifies 25 new candidates excluding their spliced variants, AtGORK and two canonical ABA receptors PYL8/RCAR3 and PYL10/RCAR4 (Supplementary File 1). Another outward rectifying channel, the *Arabidopsis thaliana* Stellar K^+^ outward rectifier, AtSKOR (At3g02850) that belongs to the same protein family as AtGORK (Shaker family), also harbours this ABA modulatory site. Interestingly, in addition to AtGORK and AtSKOR, two candidates At1g54130 and At3g14050 that synthesize guanosine tetraphosphates, have also been previously shown to respond to abscisic acid [115]. Given the biological role of ABA in promoting stomatal closure, candidates that are specifically expressed in the guard cell are of particular interest. One of them (At5g35510) is the TIR-NBS-LRR class disease resistance protein expressed in the mitochondria of guard cells. Other candidates are cytochromes (5), disease-resistance proteins (4), nucleic acid binding proteins (6), and kinase (5) and hydrolase (2) family proteins (see Supplementary File 1 for the full list of candidates). These proteins may have dual roles where e.g., proteins involved in nucleic acid binding can have their RNA/DNA interactions modulated by ABA. This initial search using a newly derived ABA motif therefore presents an exciting possibility that other molecules that harbour the motif may directly bind ABA and evoke a similar modulatory response, thus expanding our understanding of plant hormone signalling in general and ABA signalling in particular.

## 5 Summary and Outlook

Amino acid motif-based approaches, when used in tandem with structural modeling, have identified novel cellular components as well as hidden and moonlighting centers in molecules involved in different signalling pathways of the plant cell. We anticipate that this approach can be extended to other organisms especially when experimental data continue to refine existing motifs and species-specific filters are applied to tailor the motifs to a particular application. For instance, the human interleukin 1 receptor-associated kinase 3 (IRAK3) has a domain architecture similar to PSKR1 and BRI1 in *Arabidopsis thaliana*, and was reported to harbor a functional GC center [116]. Furthermore, homologs of IRAK3 in other mammalian species also harbors similar GC centers and given the role of IRAK3 in immune responses, it is crucial to elucidate the cellular and biological role(s) of such functional centers identified from this motif-based approach [116]. As motif searches continue to identify novel functional centers in other systems, the strength of this method relies on its ability to reduce misidentifications or false positives; an issue that is commonly associated to many high-throughout computational predictions [[117], [118], [119]]. In this regard, in addition to structural and molecular docking simulations, statistics and mathematical approaches can be included to the current workflow e.g., probing the topological and physic-chemical parameters (i.e., residue size, isoelectric point, hydrophobicity, amino acid enrichment) of the intermediate and flanking amino acid residues in existing search motifs, in order to provide numerical values that can help to discriminate true positives from false ones. Furthermore, increasing biological evidence of complex multifunctional proteins have provided experimental evidence in support of the search motifs while in mammalian systems, the identification of proteins with moonlighting functions is becoming an area of considerable interest [120,121]. Given the structural diversity of moonlighting proteins [122], a rapid, inexpensive and high-throughput prediction method such as this motif-based computational strategy is beneficial especially given the fact that moonlighting centers of such proteins have been implicated in human diseases [121] and are consequently targeted for the development of novel therapeutics. In one particular example, the macrophage infectivity potentiator (Mip) proteins which are virulence factors in many pathogens including *Legionella pneumophila* exert virulence by binding to collagen IV in the extracellular matrix mediated by the peptidyl-prolyl-*cis/trans*-isomerase (PPIase) domain [123]. Since the moonlighting PPIase activity of Mip is required for virulence, chemical inhibitors that specifically bind to the PPIase domain have been developed thus providing a non-immunosuppressive approach to the treatment of infections [124]. With the current encouraging evidence of identifying novel molecules involved in either synthesising or perception of second messengers, this computational motif-based approach can afford critical understanding of the cellular and biological roles of complex multifunctional molecules. Further exploitation of the current and newly designed motifs across different organisms is necessary and can lead to new and fundamental insights for example in stress signalling. Finally, given the central role the molecules described in this review, for example, in cellular homeostasis, regulation and stress responses, it is conceivable that they will be targets for biotechnological advances that will ultimately contribute to the delivery of crop plants with improved stress tolerance.

The following is the supplementary data related to this article.

Supplementary File 1 Candidate ABA modulatory sites retrieved from a PatMatch search using the ABA motif ([DE]X(7,8)RX(3,4)[DE]X(5)YX(6)H) Supplementary File 1
