# Supplementary material for: Discovery of Novel Functional Centers With Rationally Designed Amino Acid Motifs
Source: Comput Struct Biotechnol J. 2018 Feb 27;16:70–6. doi: 10.1016/j.csbj.2018.02.007 (PMC6026216; doi:10.1016/j.csbj.2018.02.007)
Supplement: Supplementary File 1 — Candidate ABA modulatory sites retrieved from a PatMatch search using the ABA motif ([DE]X(7,8)RX(3,4)[DE]X(5)YX(6)H) [file mmc1.pdf]

Supplementary File 1

Candidate ABA modulatory sites retrieved from a PatMatch search using the ABA motif ([DE]X(7,8)RX(3,4)[DE]X(5)YX(6)H)

Hits found:	33
Sequences with hits:	33
Sequences searched:	35386
Bytes searched:	14482855
Pattern:	[DE]x{7,8}Rx{3,4}[DE]x{5}Yx{6}H
Dataset searched:	TAIR10 Proteins (protein)
Download all matches as a textfile	download

List retrieved online from:
https://www.arabidopsis.org/cgi-bin/patmatch/nph-patmatch.pl
[Accessed on 21st October, 2017]

- ABA receptors or sensors
- Homolog of AtGORK
- Cytochromes
- Kinases
- Disease resistance protein
- Nucleic acid binding
- ⇨ Response to ABA

TAIR ID	Start	End	Sequence	Names, annotation and description
⇨ **AT5G37500.1**	543	569	DFYQLKSLIRSGADPNKTDYDGRSPLH	**GORK** Member of Shaker family potassium ion (K^+) channel. Mutants have increased water consumption and limited stomatal closure in response to abscisic and jasmonic acids. Forms a heteromeric K(out) channels with SKOR. Expression is suppressed during agrobacterium-induced tumor formation and increased in response to water deprivation and cold.
⇨ AT3G02850.1	560	586	DLYQLKSLIRAGGDPNKTDYDGRSPLH	SKOR Member of Shaker family potassium ion (K^+) channel. Mediates the delivery of K^+ from stelar cells to the xylem in the roots towards the shoot. mRNA accumulation is modulated by abscisic acid. K^+ gating activity is modulated by external and internal K^+. Involved in response to low potassium.
⇨ **AT5G53160.2**	101	127	DNEHILSIRIVGGDHRLKNYSSIISLH	**PYL8/RCAR3** Encodes RCAR3, a regulatory component of ABA receptor. Interacts with protein phosphatase 2Cs ABI1 and ABI2. Stimulates ABA signaling. The mRNA is cell-to-cell mobile.
⇨ **AT4G27920.1**	97	123	DNEHILGIRIVGGDHRLKNYSSTISLH	**PYL10/RCAR4** Abscisic acid sensors. Mediate ABA-dependent regulation of protein phosphatase 2Cs ABI1 and ABI2.
AT3G06150.1	193	220	DISGENWKSRPPVKDFGNGTYSFSLQVH	CYTOCHROME P450
AT5G19060.1	151	178	DLSGENWKSRPPVKDLGNGTYSLSLQIH	CYTOCHROME P450
AT3G20120.1 AT3G20120.2	131	157	EQKLDDHQDRDMMDVLLAAYGDENAEH	CYTOCHROME P450/CYP705A21
AT3G26320.1	265	290	EDFVDLLLRLEKEETVIGYGKLTRNH	CYTOCHROME P450/ CYP71B36
AT5G57260.1	265	290	DDFVDLLLRLEKEEAVLGYGKLTRNH	CYTOCHROME P450/ CYP71B10
AT2G39360.1	70	95	ESTLFQTARVFSDESSSTYRFPIEEH	CURVY1/CVY1 Protein kinase superfamily protein

AT3G57760.1<br>AT3G57760.2<br>AT3G57760.3	271	297	ELSLSSDMLRALADLFIKPYDDVRYVH	Protein kinase superfamily protein
AT1G50190.1	214	239	DELSCGVCRKLVDVNYGQYACKKGCH	Cysteine/Histidine-rich C1 domain family protein, Protein kinase C-like, phorbol ester/diacylglycerol binding
AT5G35510.1	34	59	EIQQQSDLRNATENKTEKYISDDVGH	TIR-NBS-LRR class disease resistance protein, located in mitochondria and expressed in guard cell
AT5G45260.1<br>AT5G45260.2	457	482	ETVQIERRRRLWEPWSIKYLLEYNEH	WRKY52/RRH1/SLH1<br>TIR-NBS-LRR class disease resistance protein. Confers resistance to *Ralstonia solanacearum*. Similar to NBLS-TIR resistance genes and transcription factors. Interacts with pathogen effector protein AvrPop2.
AT2G01740.1	76	101	EDIVHSMPRFGCEPDVISYNSLIDGH	Tetratricopeptide repeat (TPR)-like superfamily protein
AT3G09060.1	272	298	DDCLKIWERMKQNEREKDLYTYSSLIH	Pentatricopeptide repeat (PPR) superfamily protein
AT1G54130.1	452	477	EIHDIHGLRLIVDNEKDCYKALGVVH	RSH3<br>This gene appears to be at least partially redundant with RSH2 (At3g14050). Guanosine tetraphosphate synthesized by RSH2/RSH3 (and CRSH At3g17470) to an unknown extent can repress chloroplast gene expression and also reduce chloroplast size.
AT3G14050.1	448	473	EIHDIHGLRLIVDNEGDCYKALGVVH	RSH2<br>Response to abscisic acid and wounding
AT5G61450.1	115	142	ETNLFKLMERRGYGEEYINRYNMMTKFH	P-loop containing nucleoside triphosphate hydrolases superfamily protein
AT3G12020.1<br>AT3G12020.2	506	533	ELAYLPYKRRDMMDDEQLDLYVSVEGNH	KIN7.3/KINESIN7.3<br>P-loop containing nucleoside triphosphate hydrolases superfamily protein, involved in microtubule-based movement
AT1G36510.1	152	179	DVSTCVTIARVCFIETMPKWYYIACKAH	Nucleic acid-binding proteins superfamily
AT3G25940.1	88	114	ELVYTTRQTRSADEGQTTYYTCPNCAH	TFIIB zinc-binding protein, DNA binding
AT4G22680.1	237	262	DFPIWSPERINDEKMFLDYCQDFGVH	MYB85<br>Encodes a putative transcription factor (MYB85), DNA binding
AT1G28180.1	199	224	EEMNERDWRIFKEDFNISYRGSKIPH	DEAD-box ATP-dependent RNA helicase-like protein, RNA binding
AT2G13500.1	187	212	ESGETALIRFQYERLRRIYSCFRFTH	Ta11-like non-LTR retrotransposon
AT5G42390.1	1150	1176	DRAKRTLLMRHEAELKSNAYWLNLLAH	SPP<br>Encodes a chloroplast-localized metalloprotease that is essential for embryo development. Mutants do not progress normally beyond the 16-cell stage and mRNA is cell-to-cell mobile.
AT5G43850.1	139	166	DASNYIKLMRLFVGEPVWTPYNRPQEEH	ARD4<br>RmlC-like cupins superfamily protein
AT5G47650.1	249	275	DYEGFTPLRVSAPDQQGNLYYNTRDLH	NUDT2/NUDX2<br>Encodes an ADP-ribose pyrophosphatase that confers enhanced tolerance to oxidative stress.

* AT1G54130.1 and AT3G14050.1 are also annotated protein kinases
